# Model selection and robust inference of mutational signatures using Negative Binomial non-negative matrix factorization

**DOI:** 10.1186/s12859-023-05304-1

**Published:** 2023-05-08

**Authors:** Marta Pelizzola, Ragnhild Laursen, Asger Hobolth

**Affiliations:** Department of Mathematics, Aarhus University, Aarhus, Denmark

**Keywords:** Cancer genomics, Cross-validation, Model checking, Model selection, Mutational signatures, Negative Binomial, Non-negative matrix factorization, Poisson, 92-08, 92-10, 62-08

## Abstract

**Background:**

The spectrum of mutations in a collection of cancer genomes can be described by a mixture of a few mutational signatures. The mutational signatures can be found using non-negative matrix factorization (NMF). To extract the mutational signatures we have to assume a distribution for the observed mutational counts and a number of mutational signatures. In most applications, the mutational counts are assumed to be Poisson distributed, and the rank is chosen by comparing the fit of several models with the same underlying distribution and different values for the rank using classical model selection procedures. However, the counts are often overdispersed, and thus the Negative Binomial distribution is more appropriate.

**Results:**

We propose a Negative Binomial NMF with a patient specific dispersion parameter to capture the variation across patients and derive the corresponding update rules for parameter estimation. We also introduce a novel model selection procedure inspired by cross-validation to determine the number of signatures. Using simulations, we study the influence of the distributional assumption on our method together with other classical model selection procedures. We also present a simulation study with a method comparison where we show that state-of-the-art methods are highly overestimating the number of signatures when overdispersion is present. We apply our proposed analysis on a wide range of simulated data and on two real data sets from breast and prostate cancer patients. On the real data we describe a residual analysis to investigate and validate the model choice.

**Conclusions:**

With our results on simulated and real data we show that our model selection procedure is more robust at determining the correct number of signatures under model misspecification. We also show that our model selection procedure is more accurate than the available methods in the literature for finding the true number of signatures. Lastly, the residual analysis clearly emphasizes the overdispersion in the mutational count data. The code for our model selection procedure and Negative Binomial NMF is available in the R package SigMoS and can be found at https://github.com/MartaPelizzola/SigMoS.

**Supplementary Information:**

The online version contains supplementary material available at 10.1186/s12859-023-05304-1.

## Introduction

Somatic mutations occur relatively often in the human genome and are mostly neutral. However, the accumulation of harmful mutations in a genome can lead to cancer. The summary of somatic mutations observed in a tumor is called a mutational profile and can often be associated with factors such as aging [1], UV light [2] or tobacco smoking [3]. A mutational profile is thus a mixture of mutational processes that are represented by mutational signatures. Several signatures have been identified from the mutational profiles and associated with different cancer types [4, 5]. The importance of mutational signatures thus lies in their association with the mutational processes causing cancer. Having more insights into the causes of cancer is a prerequisite for better understanding the role that genetics plays in the development of the disease and eventually also for discovering potential treatment.

A common strategy to derive the mutational signatures is non-negative matrix factorization [6–8]. Different approaches to estimate the signature and the exposure matrices from mutational count data have been extensively described in [9, 10].

Non-negative matrix factorization (NMF) is a factorization of a given matrix $V \in {\mathbb{N}}_0^{\text{N} \times \text{M}}$ into the product of two non-negative matrices $W \in {\mathbb{R}}_+^{\text{N} \times \text{K}}$ and $H \in {\mathbb{R}}_+^{\text{K} \times \text{M}}$ such that

$$\begin{aligned}V \approx WH.\end{aligned}$$

The rank K of the lower-dimensional matrices *W* and *H* is much smaller than N and M.

In cancer genomics, the mutational matrix *V* contains the mutational counts for different patients, also referred to as mutational profiles. The number of rows N is the number of patients and the number of columns M is the number of different mutation types. In this paper we use the single-base-substitution-96 mutational context [11] where $\text{M} = 96$ (corresponding to the 6 base mutations when assuming strand symmetry times the 4 flanking nucleotides on each side, i.e. $4 \cdot 6 \cdot 4 = 96$). The matrix *H* consists of K mutational signatures defined by probability vectors over the different mutation types. In the matrix *W*, each row contains the weights of the signatures for the corresponding patient. In this context, the weights are usually referred to as the exposures of the different signatures.

To estimate *W* and *H* we need to choose a model and a rank K for the data *V*. These two decisions are highly related as the optimal rank of the data *V* is often chosen by comparing the fit under a certain model for many different values of K. The optimal K is then found using a model selection procedure such as Akaike Information Criterion (AIC), Bayesian Information Criterion (BIC) or similar approaches described in “Estimating the number of signatures” section. Most methods used in the literature [6, 12, 13] for choosing the rank are based on the likelihood value, which depends on the assumed model. For mutational counts the usual model assumption is the Poisson distribution [6]

1 $$\begin{aligned} V_{nm} \sim \text{Po}((WH)_{nm}), \end{aligned}$$

where *W* and *H* are estimated using the algorithm from [14] that minimizes the generalized Kullback–Leibler divergence. The algorithm is equivalent to maximum likelihood estimation, as the negative log-likelihood function for the Poisson model is equal to the generalized Kullback–Leibler up to an additive constant. We observe that this model assumption is often inadequate. In particular, we observe overdispersion in the mutational counts, i.e. a situation where the variance in the data is greater than what is expected under the assumed model. This is a well known issue when modeling count data in biology [15].

We therefore suggest using a model where the mutational counts follow a Negative Binomial distribution that has an additional parameter to explain the overdispersion in the data. In recent years, this model is becoming more popular to model the dispersion in mutational counts [16, 17]. The Negative Binomial NMF is discussed in [18], where it is applied to recommender systems, and it has recently been used in the context of cancer mutations in [19–21]. In Lyu et al. [20] a supervised Negative Binomial NMF model is applied to mutational counts from different cancers which uses cancer types as metadata. Their aim is to obtain signatures with a clear etiology, which could be used to classify different cancer types. Vöhringer et al. [21] extends the analysis by including several genomic features and uses tensors instead of the mutational count matrix to account for the different features. Lastly, [19] applies Bayesian inference to extract mutational signatures and provide different probabilistic models for the signatures. Among the models implemented in this method also the Negative Binomial model is considered as a natural extension of the Poisson model.

For mutational count data, we extend the Negative Binomial NMF model by including patient specific dispersion which has not been included in the aforementioned works using the Negative Binomial model. The extended model is referred to as NB_N_-NMF, where N is the number of dispersion parameters (equivalent to the number of patients). We investigate when and why NB_N_-NMF is more suitable for mutational counts than the usual Poisson NMF (Po-NMF). In particular we evaluate the goodness of fit for mutational counts using a residual-based approach. Despite the recent efforts, we still believe, as it has also been mentioned in [22], that a great amount of research has been focusing on improving the performance of NMF algorithms given an underlying model and less attention has been directed to the choice of the underlying model given the data and application.

Since the number of signatures depends on the chosen distributional assumption, we suggest using NB_N_-NMF and we also propose a novel model selection framework to choose the number of signatures. We show that our model selection procedure is more robust toward inappropriate model assumptions compared to classical methods (AIC and BIC) and other methods currently used in the literature such as $\texttt{SigProfilerExtractor}$ [23], $\texttt{SparseSignatures}$ [8], $\texttt{SigneR}$ [13], $\texttt{sigfit}$ [19], and $\texttt{SignatureAnalyzer}$ [24]. We use both simulated and real data to validate our proposed model selection procedure against other methods. We chose one classical data set and analyze it in “Breast cancer data” section and a larger data set from prostate cancer (Fig. 5). The latter is a subset of the available data from the Pan-Cancer Analysis of Whole Genomes (PCAWG) database [25], thus it corresponds to one of the largest available data sets for a single cancer type.

In comparison to the results published in [20] and in [21], our work is not exploiting the information coming from different cancer types or from different genomic features. However, we provide a patient specific dispersion component to account for the high variance between patients and derive the update steps for parameter estimation in the NB_N_-NMF. Furthermore, we propose a model selection procedure which proves to be robust to model misspecification.

We have implemented our methods in the R package SigMoS (Signatures Model Selection) that includes NB_N_-NMF and the model selection procedure. The R package is available at https://github.com/MartaPelizzola/SigMoS. The package also contains the simulated and real data used in this paper.

## Results

In this section we describe the results of our approach on both simulated and real data. Details on the method are provided in “Methods” section. In short, we propose a Negative Binomial model applied to mutational count data with a patient specific dispersion coefficient. The matrices *W* and *H* are estimated with a majorization–minimization (MM) procedure, and we propose to use Negative Binomial maximum likelihood estimation (MLE) for estimating the dispersion parameters. Additionally, we introduce a new algorithm based on cross-validation to estimate the number of signatures for a given data set.

For simulated data we present a study on Negative Binomial simulated data with different levels of dispersion where results from AIC, BIC, $\texttt{SigProfilerExtractor}$ [23], $\texttt{SparseSignatures}$ [8], $\texttt{SigneR}$ [13], $\texttt{sigfit}$ [19] and $\texttt{SignatureAnalyzer}$ [24] are compared with our proposed model selection procedure. These results are discussed in “Simulation study” section, where we show that our method performs well and is robust to model misspecification. Our method is applied to the 21 breast cancer patients from [6] in “Breast cancer data” section, and to 286 prostate cancer patients from [25] in “Prostate cancer data” section. The goodness of fit of the different models are evaluated using a residual analysis that shows a clear overdispersion with the Poisson model. The use of residual plots to evaluate the goodness of fit is a common strategy in statistics; some examples can be found in [26, 27].

### Simulation study

We simulated our data following the procedure of [8] using the signatures from [5]. We simulated 100 data sets for each scenario and varied the number of patients, the number of signatures and the model for the noise in the mutational count data. We considered 20, 100 and 300 patients and either 5 or 10 signatures following [28] which states that the number of common signatures in each organ is usually between 5 and 10. For each simulation run we use signature 1 and 5 from [5], as they have been shown to be shared across all cancer types, and then we sample at random three or eight additional signatures from this set. The exposures are simulated from a Negative Binomial model with mean 6000 and dispersion parameter 1.5 as in [8]. This choice is based on estimates from the real data in [29]. The mutational count data is then generated as the product of the exposure and signature matrix. Lastly, Poisson noise, Negative Binomial noise with dispersion parameter $\alpha \in \{10, 200\}$ or uniformly sampled in [10, 500] are added to the mutational counts. The values of the patient specific dispersion are inspired from the data set in “Breast cancer data” section. A lower $\alpha$ is associated with higher dispersion, however the actual level of dispersion associated to a given $\alpha$ value depends on the absolute mutational counts as can be seen from the variance in Eq. (5). Therefore it is not possible to directly compare these values with the ones estimated for the real data.

#### Simulation results

**Fig. 1:**
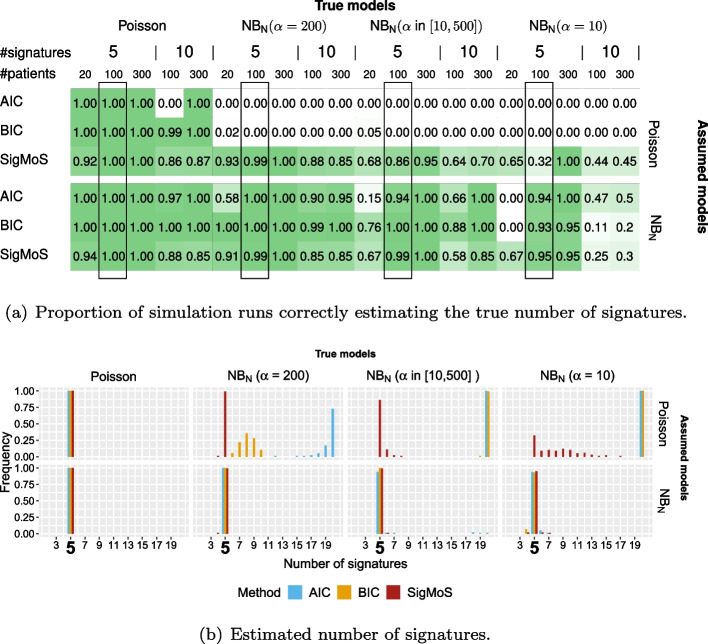
Results from AIC, BIC, and SigMoS based on Po-NMF and NB_N_-NMF using simulated data. Each method is applied on different simulated data sets for four different types of noise: Poisson and Negative Binomial with dispersion parameter $\alpha = 10, 200$ and $\alpha \sim U(10,500)$. **a** The proportion of simulation runs where the number of signatures is correctly estimated. The true number of signatures varies in $\{5,10\}$ and the number of patients in $\{20,100,300\}$. The rectangular boxes highlight the results shown in **b**. The results are based on 100 simulation runs for scenarios with 20 and 100 patients and on 20 simulation runs for scenarios with 300 patients. **b** The estimated number of signatures in the range from 2 to 20 for 100 patients, where the true number of signatures is five

The effect of the model assumption on the estimated number of signatures using AIC, BIC (see Eqs. (14) and (15)) and SigMoS as model selection procedures is shown in Fig. 1. Figure 1a summarizes results for all simulation studies and for each study. This figure displays the proportion of scenarios where the true number of signatures is correctly estimated from the different methods: the darker the green color the higher is this proportion. This shows that our proposed approach is estimating the number of signatures accurately and is much more robust to model misspecifications compared to AIC and BIC. For example, when the true model has a small dispersion of $\alpha = 200$ and the Poisson model is assumed, the difference between the performance of SigMoS and of AIC and BIC is already substantial. Here, AIC and BIC are never estimating the true number of signatures correctly, whereas our SigMoS procedure estimates the correct number of signatures in most cases ($\ge 85\%$). The table also shows that the higher the dispersion in the model, the harder it is to estimate the true number of signatures even when the correct model is specified.

Figure 1b depicts the actual estimated number of signatures in the range from 2 to 20 for the 100 data sets with 5 signatures and 100 patients. This clearly shows that the higher the overdispersion in the model, the more is the number of signatures overestimated. Assuming Poisson in the case of $\alpha = 200$ we see that AIC is already overestimating the number of signatures. Here, these additional signatures are needed to explain the noise that is not accounted for by the Poisson model. Having an even higher overdispersion makes both AIC and BIC highly overestimate the number of signatures to a value that is plausibly much higher than 20. Even high overdispersion does not influence our SigMoS procedure in the same way and our approach is still estimating the true number of signatures for a large proportion of the scenarios. Assuming the Negative Binomial model all of the three methods have a really high performance, as the Negative Binomial accounts for both low and high dispersion.

In the simulation study from Fig. 1b we also consider the accuracy of the MLE for the $\alpha$ value in the two scenarios where each patient has the same $\alpha$. Our approach estimates the true $\alpha$ with high accuracy when the dispersion is high i.e.  ${\hat{\alpha }} \in [9.21, 11.78]$ for $\alpha = 10$, $\alpha$ is slightly overestimated when the dispersion is low: for $\alpha = 200$ we find ${\hat{\alpha }} \in [225.8, 292.7]$. However, according to Fig. 1b this small bias does not affect the performance of our model selection procedure.

#### Method comparison

Several methods have been proposed in the literature for estimating the number of signatures in cancer data. In the following we present the results of a comparison between our method and four commonly used methods in the literature: $\texttt{SigProfilerExtractor}$ [23], $\texttt{SparseSignatures}$ [8], $\texttt{SignatureAnalyzer}$ [24], $\texttt{sigfit}$ [19], and $\texttt{SigneR}$ [13]. $\texttt{SigProfilerExtractor}$ [23] extracts mutational signatures by applying NMF to 100 normalized Poisson resampled input matrices for different values for the number of signatures. The number of mutational signatures is then estimated by evaluating the stability of mutational signatures and choosing the solution with the lowest number of signatures among the stable solutions that describe the data well. $\texttt{SparseSignatures}$ [8] provides an alternative cross-validation approach where the test set is defined by setting 1% of the entries in the count matrix to 0. Then NMF is iteratively applied to the modified count matrix and the entries are updated at each iteration. The resulting signature and exposure matrices are used to predict the entries of the matrix corresponding to the test set. $\texttt{SignatureAnalyzer}$ [24], on the other hand, proposes a procedure where a Bayesian model is used and maximum a posteriori estimates are found with a majorize-minimization algorithm. $\texttt{sigfit}$ [19] presents an R package providing different options for extracting and refitting signatures and exposures by Bayesian inference under different models. They propose a framework where a Multinomial, Normal, Poisson or Negative Binomial model (with mutation type specific dispersion parameter) can be used. The number of signatures is estimated using the elbow method by looking at changes in the accuracy of re-estimating the data with the extracted signatures and exposures. In our comparison we use the Poisson and Negative Binomial models within the $\texttt{sigfit}$ package and refer to them as $\mathtt {sigfit\text{- }Po}$ and $\mathtt {sigfit\text{- }NB}$. Lastly, with $\texttt{SigneR}$ [13] an empirical Bayesian approach based on BIC is used to estimate the number of mutational signatures.

For our method comparison, we run all methods on the simulated data from Fig. 1b. For each method and simulation setup we only allow the number of signatures to vary from two to eight due to the long running time of some of these methods.

**Fig. 2:**
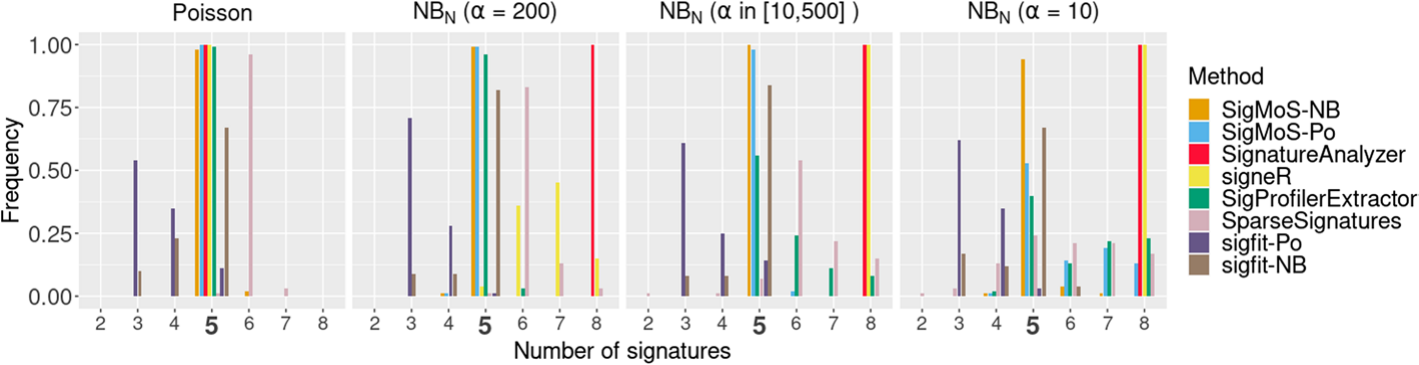
Method comparison using simulated data. Each method is applied on the data sets from Fig. 1b and, for each data set, the value of the estimated number of signatures is kept. We test values for the number of signatures from two to eight for Poisson noise and Negative Binomial noise with $\alpha = \{10, 200 \}$, and a patient specific dispersion parameter $\alpha \sim U(10,500)$

Figure 2 shows that, when Poisson data are simulated almost all methods have a very good performance and can recover the true number of signatures in most of the simulations. The poor performance of $\texttt{SparseSignatures}$ could be affected by not having a fixed background signature. Indeed, the improved performance of $\texttt{SparseSignatures}$ when a background signature is included has also been shown in [8]. $\mathtt {sigfit\text{- }Po}$ is based on a more heuristic method and tends to underestimate the true number of signatures. When Negative Binomial noise is added to the simulated data with a moderate dispersion ($\alpha = 200$), $\mathtt {sigfit\text{- }Po}$, $\texttt{SignatureAnalyzer}$ and $\texttt{SigneR}$ have low power emphasizing the importance of correctly specifying the distribution for these methods, whereas our proposed approach (regardless of the distributional assumption), $\mathtt {sigfit\text{- }NB}$, $\texttt{SigProfilerExtractor}$ and $\texttt{SparseSignatures}$ maintain good power. For patient specific dispersion also the power of $\texttt{SparseSignatures}$ and $\texttt{SigProfilerExtractor}$ decreases. Lastly, the power of $\mathtt {sigfit\text{- }NB}$ decreases for high dispersion ($\alpha = 10$): here the distributional assumptions are correctly specified, however this is a heuristic approach to estimate the number of signatures which tends to be less precise than SigMoS. Indeed, good performance is achieved with our proposed approach even under high dispersion if the correct distribution is assumed. These results demonstrate that SigMoS is accurate for detecting the correct number of signatures and it performs well also in situations with overdispersion compared to other methods.

**Fig. 3:**
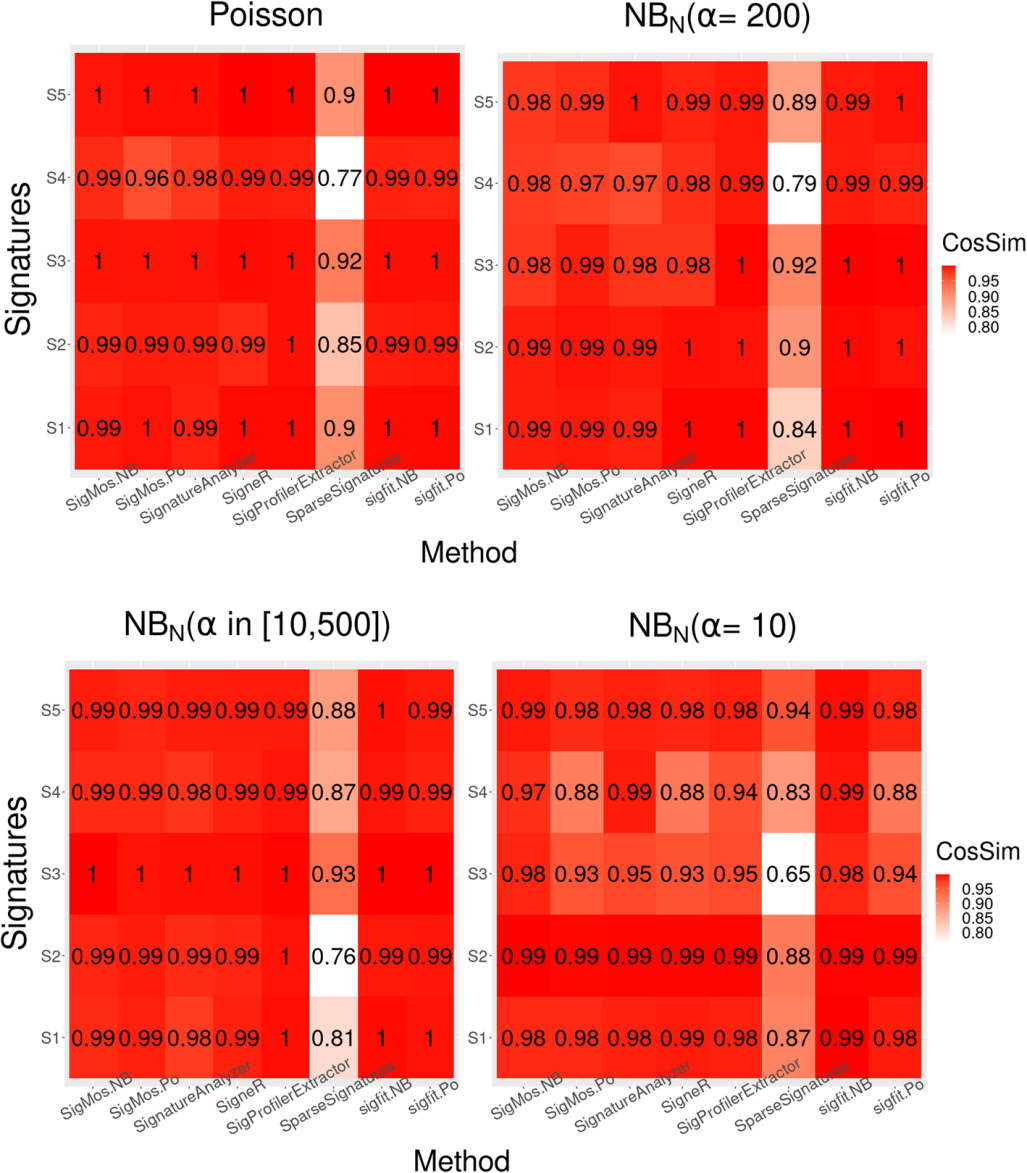
Quality of estimated signatures using simulated data. Each method is applied on 10 randomly sampled data sets from Fig. 1b and, for each data set, the value of the estimated number of signatures is fixed to 5. We show the quality of the estimated signatures measured by cosine similarity for all methods with Poisson noise and Negative Binomial noise with $\alpha = 10, 200$, and a patient specific dispersion parameter $\alpha \sim U(10,500)$

For this set of simulations we also checked the quality of the estimated signatures. We sampled 10 runs for each scenario from Fig. 2 and calculated the cosine similarity between the estimated signatures and the true ones used for simulations. The results for all methods are shown in Fig. 3 where we display the average cosine similarity over 10 runs for each method and each scenario. For this study we fixed the number of signatures to five for all methods, which may favour methods such as $\texttt{SignatureAnalyzer}$, $\texttt{sigfit}$ or $\texttt{SigneR}$ that usually overestimate the number of signatures. Nonetheless, these results also show that SigMoS combined with the Negative Binomial model and $\mathtt {sigfit\text{- }NB}$ are the methods that are able to retain the highest accuracy also with high levels of overdispersion (namely $\alpha = 10$). $\texttt{SigProfilerExtractor}$ and $\texttt{SigneR}$ also show good accuracy especially when the overdispersion is low and under the Poisson model. These results, combined with those in Fig. 2, show that for real data where the variance may be higher than the one accounted for under the Poisson model, using a Negative Binomial model is essential. Indeed, this distributional assumption leads to high accuracy in the estimated signatures and SigMoS combined with the Negative Binomial model is able to maintain high accuracy and also correctly infer the true number of signatures.

We additionally compared our method to an independent set of simulated data from [30]. Here, the authors propose an alternative cross-validation procedure for estimating the number of signatures and describe a method comparison where $\texttt{SigProfilerExtractor}$, $\texttt{SignatureAnalyzer}$ and $\texttt{SigneR}$ are included. We considered their 20 simulated data sets comprising of 200 patients and 9 signatures each and we run SigMoS under both the Negative Binomial and the Poisson model. The signatures used for this set of simulations have been taken from the PCAWG breast cancer study [4] where two pairs of signatures are highly similar, namely signatures SBS1 and SBS5 as well as SBS2 and SBS13, and their exposures have been resampled jointly when generating the data. It is not surprising that our method often estimates less than 9 signatures (7 or 8 signatures are reconstructed in most of the scenarios). We compared these results to the ones in [30] where a method based on cross-validation is proposed to estimate the number of signatures. Here, an extensive method comparison is available showing the accuracy in estimating the true signatures. We provide similar results in Additional file 1: Figs. S1 and S2 where our method is run with Po-NMF and NB_N_-NMF. Comparing these results to Fig. S9 in [30], we can see that most methods tend to estimate less than 9 signatures and that the accuracy of the signatures estimated by SigMoS is always higher or comparable to the ones estimated by the other methods.

These results indicate that our proposed approach is robust to different simulation set ups, has very good performance on a wide range of scenarios, and provides more accurate estimates of the underlying number of signatures and of the actual mutational signatures when compared to other methods available in the literature, suggesting that it will also be robust when applied to real data. Computational cost results for our method in terms of memory usage and time until convergence as a function of the number of patients are available in Additional file 1: Section S2. SigMoS runs on a standard laptop with Intel Core i7 processor in less than a few minutes and uses less than 25 gigabase of memory for data sets with up to 500 patients and 5 signatures. Both memory consumption and running time increase linearly with the number of patients, but even large data sets can be run fairly quickly on a standard laptop (for 1000 patients SigMoS used up to 100 GB and the running time went up to 7 min for the slowest scenarios).

### Breast cancer data

This data set consists of the mutational counts from the 21 breast cancer patients that has previously been described and analyzed in several papers [6, 7, 12]. The data can be found through the link ftp://ftp.sanger.ac.uk/pub/cancer/AlexandrovEtAl from [11] and have been extensively analyzed in [4].

**Fig. 4:**
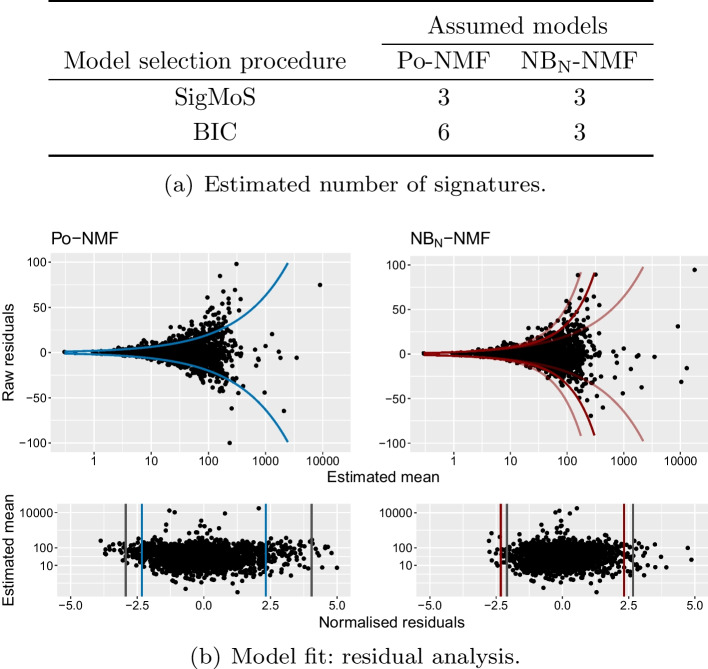
Results for Po-NMF and NB_N_-NMF applied to a data set with 21 breast cancer patients. **a** The optimal number of signatures estimated from SigMoS and BIC when using Po-NMF and NB_N_-NMF. **b** The residual plots for Po-NMF and $\hbox{NB}_\text{N}$-NMF when assuming the estimated number of signatures from SigMoS i.e. 3 signatures in both cases. The lines in the top plot correspond to two times the expected variance under the chosen distributional assumption. As the $\hbox{NB}_\text{N}$-NMF holds 21 different expected variances, we have chosen to plot the median, minimum and maximum variance among the 21. The second plots show the normalized residuals. The vertical blue and red lines depict the theoretical quantiles and the gray lines show the observed quantiles

In Fig. 4a, we have applied SigMoS and BIC to choose the number of signatures for both Po-NMF and NB_N_-NMF. We have included the BIC to compare with the SigMoS method as it provides similar results to the state-of-the-art methods. SigMoS indicates to use three signatures for both methods. This is in line with the results of our simulation study, where we show that our model selection is robust to model misspecification. According to BIC, six signatures are needed for Po-NMF whereas only three signatures should be used with NB_N_-NMF which emphasizes the importance of a correct model choice when using BIC. In this framework and in “Prostate cancer data” section we compared SigMoS to BIC, as Fig. 1 shows that this is more robust than AIC. BIC is also often used as model selection criteria in the analysis of real data sets in the literature. We refer to “Method comparison” section for comparisons with other state-of-the-art methods.

For three signatures we show in Fig. 4b the corresponding raw residuals $R_{nm} = V_{nm} - (WH)_{nm}$ to determine the best fitting model. The residuals are plotted against the expected mean $(WH)_{nm}$, as the variance in both the Poisson and Negative Binomial model depends on this value. The colored lines in the residual plots correspond to $\pm 2\sigma$ for the Poisson and the Negative Binomial distribution, respectively. The variance $\sigma ^2$ can be derived from Eq. (5) for the Negative Binomial model and is equal to the mean for the Poisson model.

For Po-NMF we observe a clear overdispersion in the residuals, which suggests to use a Negative Binomial model. In the residual plot for the $\hbox{NB}_\text{N}$-NMF we see that the residuals have a much better fit to the variance structure, which is indicated by the colored lines. The quantile lines in the lower panel with normalized residuals again show that the quantiles from the $\hbox{NB}_\text{N}$-NMF are much closer to the theoretical ones, suggesting that the Negative Binomial model is better suited for this data. The patient specific dispersion is very diverse in this data as the $\alpha$ values for the first 20 patients are between 16 (very high dispersion) and 550 (moderate dispersion) and the last patient has $\alpha _{21} = 26083$.

We compare the signatures found by our method to the available signatures in the COSMIC database [5] downloaded from https://cancer.sanger.ac.uk/cosmic. We find that our three reconstructed signatures are similar to signatures SBS1, SBS2, SBS3. The corresponding cosine similarities are reported in Table 1 and show high similarity between our reconstructed signatures and the ones from the COSMIC database especially for SBS2 and SBS3. Indeed, a cosine similarity of 0.8 has been used as threshold in [31] to group similar signatures, suggesting that SigMoS is able to identify relevant signatures in the COSMIC database. According to the results in [4] SBS1 and SBS2 are found across most cancer types and a large proportion of breast cancer samples showing these two signatures has been found. SBS3 has also been found in a large proportion of breast cancer samples and it also has high mutational burden in breast cancer tumors. SBS3 has also been associated to the BRCA1/2 mutation [4]. The validation of our signatures with the COSMIC database shows that in this case SigMoS can correctly infer signatures that have been proved to be strongly associated with breast cancer.

**Table 1:** Cosine similarity for the breast cancer data set between the signatures extracted by SigMoS and the ones in the COSMIC database

	SBS1	SBS2	SBS3
Po-NMF	0.65	0.76	0.79
$\hbox{NB}_\text{N}$-NMF	0.62	0.76	0.80

The COSMIC signature with the highest cosine similarity is shown for each signature estimated by SigMoS

### Prostate cancer data

We also considered a more recent data set from the Pan-Cancer Analysis of Whole Genomes (PCAWG) database [25] where 2782 patients from different cancer types are available. The mutational counts from the full PCAWG database can be found at https://www.synapse.org/#!Synapse:syn11726620. From this data set, we extracted mutational counts for all the 286 prostate cancer patients and used them directly for our analysis.

**Fig. 5:**
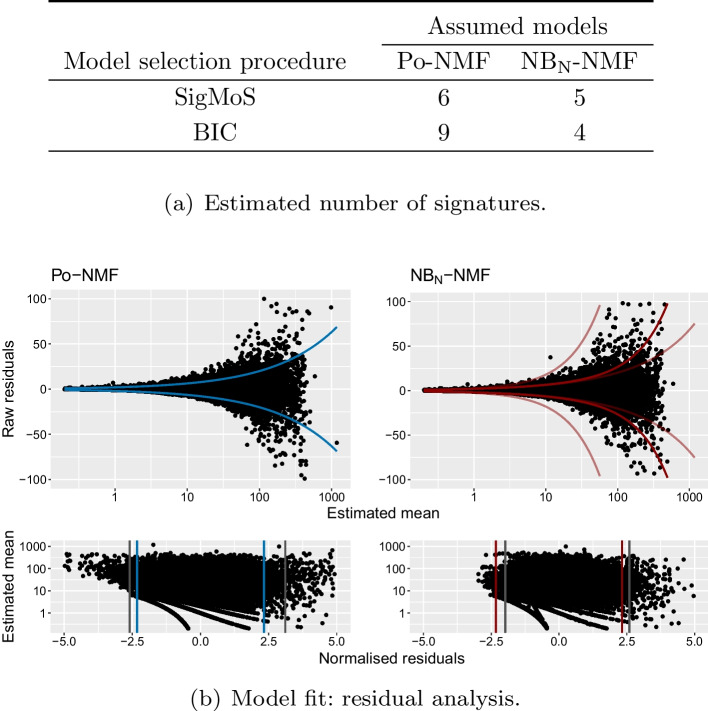
Results for Po-NMF and $\hbox{NB}_\text{N}$-NMF applied to a data set with 286 prostate cancer patients from the PCAWG database [25]. **a** The optimal number of signatures estimated from SigMoS and BIC when using Po-NMF and $\hbox{NB}_\text{N}$-NMF. **b** The residual plots for Po-NMF and $\hbox{NB}_\text{N}$-NMF when assuming the estimated number of signatures from SigMoS i.e. 5 and 6 signatures. The lines in the first plot correspond to two times the expected variance under the chosen distributional assumption. For $\hbox{NB}_\text{N}$-NMF, the colored lines in the top plot show the median, minimum and maximum variance among the patients. The bottom plots show the normalized residuals. The vertical blue and red lines depict the theoretical quantiles and the gray lines the observed quantiles

We chose again both the Poisson and Negative Binomial as underlying distributions for the NMF and in both cases we applied SigMoS for determining the number of signatures. We present the results in Fig. 5. Figure 5a shows again that our model selection procedure is more stable under model misspecification compared to BIC: the estimated number of signatures is changing from 9 to 4 between the two model assumptions for BIC, but only from 6 to 5 for SigMoS. As for Fig. 4b, the residuals in Fig. 5b show that the $\hbox{NB}_\text{N}$-NMF model provides a much better fit to the data than the Po-NMF. The estimated values for the patient specific dispersion are $\alpha _n \in [1.4,4279]$ with a median of 140 (corresponding to a quite large dispersion).

As for the previous section we compare our reconstructed signatures with the ones in the COSMIC database. Table 2 shows the cosine similarity between the signatures extracted by SigMoS and the most similar ones from the COSMIC repository. Here, $\hbox{NB}_\text{N}$-NMF provides much better results in terms of signatures estimation showing the importance of accounting for overdispersion. Indeed, $\hbox{NB}_\text{N}$-NMF finds signatures SBS1, SBS5, SBS8, SBS18, SBS37. These signatures are all largely present in prostate cancer either for their presence in many prostate tumor samples or for their contribution in terms of number of mutations per tumor or for both reasons combined. On the contrary, signatures SBS6 and SBS36 are not found in prostate cancer, showing that $\hbox{NB}_\text{N}$-NMF is more accurate.

**Table 2:**
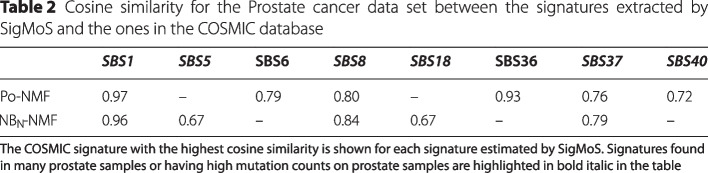
Cosine similarity for the Prostate cancer data set between the signatures extracted by SigMoS and the ones in the COSMIC database

The COSMIC signature with the highest cosine similarity is shown for each signature estimated by SigMoS. Signatures found in many prostate samples or having high mutation counts on prostate samples are highlighted in colour in the table

## Discussion

Mutational profiles from cancer patients are a widely used source of information and NMF is often applied to these data in order to identify signatures associated with cancer types. We propose a new approach to perform the analysis and signature extraction from mutational count data where we emphasize the importance of validating the model using residual analysis, and we propose a robust model selection procedure.

We use the Negative Binomial model as an alternative to the commonly used Poisson model as the Negative Binomial can account for the high dispersion in the data. As a further extension of this model, we allow the Negative Binomial to have a patient specific variability component to account for heterogeneous variance across patients.

We propose a model selection approach for choosing the number of signatures. As we show in “Simulation study” section this method works well with both Negative Binomial and Poisson data, and it is a robust procedure for choosing the number of signatures. We note that the choice of the divergence measure for the *cost* function in Algorithm 2 is not trivial and may favor one or the other model and thus a comparison of the costs between different NMF methods is not possible. For example, in our framework, we use the Kullback–Leibler divergence which would favor the Poisson model. This means that a direct comparison between the cost values for Po-NMF and $\hbox{NB}_\text{N}$-NMF is not feasible. To check the goodness of fit and choose between the Poisson model and the Negative Binomial model we propose to use the residuals instead.

In Additional file 1: Section S4, we investigated the role of the cost function in our model selection by including the Frobenius norm and Itakura–Saito (IS) [32] divergence measure from [33], where the authors propose a fast implementation of the NMF algorithm with general Bregman divergence. In this investigation the cost function did not influence the optimal number of signatures. The only difference was how the cost values differed among the NMF methods, as each cost function favored the models differently. Therefore we chose to use the Kullback–Leibler divergence and compared the methods with the residual analysis.

Less signatures are found when accounting for overdispersion with the Negative Binomial model. Indeed, there is no need to have additional signatures explaining noise, which we assume is the case for the Poisson model. We show that the Negative Binomial model is more suitable and therefore believe the corresponding signatures are more accurate. This can be helpful when working with mutational profiles for being able to better associate signatures with cancer types and for a clearer interpretation of the signatures when analyzing mutational count data. For example, the recent results in [28] use a large data set with several different cancer types and show that there exists a set of common signatures that is shared across organs and a set of rare signatures that are only found with a sufficiently large sample size. To recover the common signatures the patients with unusual mutational profiles were excluded as they are introducing additional variance in the signature estimation procedure. We speculate that changing the Poisson assumption in this approach with the Negative Binomial distribution could provide a simpler and more robust way to extract common signatures. Indeed, the Negative Binomial model allows for more variability in the data and our simulation results and residual plots in “Results” section show that the Negative Binomial distribution is beneficial for stable signature estimation. In this work we have focused on single base substitutions, but the Negative Binomial NMF can be highly beneficial also for analyzing indels or other variant types. In [4] they discuss that mutational matrices corresponding to indels harbor more variation which means that more flexible models than the Poisson are needed in this situation.

The workflow for analyzing the data, and the procedures in Algorithms 1 and 2 are available in the R package SigMoS at https://github.com/MartaPelizzola/SigMoS.

## Methods

This section is structured as follows: in “Negative Binomial model for mutational counts” section we describe the Negative Binomial model applied to mutational count data. Then we propose an extension where a patient specific dispersion coefficient is used. The majorization–minimization (MM) procedure for patient specific dispersion $\{ \alpha _1, \ldots , \alpha _N \}$ can be found in “Patient specific NB_N_-NMF” section. In our application, we propose to use Negative Binomial maximum likelihood estimation (MLE) for $\alpha$ and $\{ \alpha _n: 1 \le n \le N \}$ instead of the grid search adopted in [18]. The pseudocode shown in the initial steps of Algorithm 1 describes this approach for patient specific dispersion. For shared dispersion among all patients and mutation types we simply set $\alpha = \alpha _1 = \cdots = \alpha _N$ in Algorithm 1. Lastly, in “Estimating the number of signatures” section we describe our proposed algorithm to estimate the number of signatures.

### Negative Binomial model for mutational counts

In this section we argue why the Negative Binomial model in [18] is a natural model for the number of somatic mutations in a cancer patient. We start by illustrating the equivalence of the Negative Binomial to the more natural Beta-Binomial model as a motivation for our model choice.

Assume a certain mutation type can occur in $\tau$ triplets along the genome with a probability *p*. Then it is natural to model the mutational counts with a binomial distribution [34, 35]

2 $$\begin{aligned} V_{nm} \sim \text{Bin}(\tau ,p). \end{aligned}$$

However, [36] observed that the probability of a mutation varies along the genome and is correlated with both expression levels and DNA replication timing. We therefore introduce the Beta-Binomial model

3 $$\begin{aligned} \begin{aligned} V_{nm}|&p \sim \text{Bin}( \tau , p) \\&p \sim \text{Beta}(\alpha , \beta ), \end{aligned} \end{aligned}$$

where the beta prior on the probability *p* models the heterogeneity of the probability of a mutation for the different mutation types due to the high variance along the genome. As *p* follows a Beta distribution, its expected value is $\mathbb{E}[p] = {\alpha }/{(\alpha + \beta )}$. For mutational counts, the number of triplets $\tau$ is extremely large and the probability of mutation *p* is very small. In the data described in [36] there are typically between 1 and 10 mutations per megabase with an average of 4 mutations per megabase ($\tau \approx 10^6$). This means $\mathbb{E}[p] = {\alpha }/{(\alpha + \beta )} \approx 4 \cdot 10^{-6}$ and thus, for mutational counts in cancer genomes we have that $\beta>> \alpha$. As $\tau$ is large and *p* is small, the Binomial model is very well approximated by the Poisson model $\text{Bin}(\tau , p) \backsimeq \text{Pois}(\tau p)$. This distributional equivalence of Poisson and Binomial when $\tau$ is large and *p* is small is well known. This also means that the models (1) and (2) are approximately equivalent with $\tau p = (WH)_{nm}$.

The Beta and Gamma distributions are also approximately equivalent in our setting. Indeed, as $\beta>> \alpha$, the Beta density can be approximated by the Gamma density in the following way

$$\begin{aligned} \frac{p^{\alpha - 1}(1-p)^{\beta - 1}}{B(\alpha , \beta )}&= \frac{p^{\alpha - 1}}{\Gamma (\alpha )} (\beta -1+\alpha )(\beta -1 + (\alpha -1)) \cdots (\beta -1) (1-p)^{\beta - 1} \\&\approx \frac{p^{\alpha - 1}}{\Gamma (\alpha )} \beta ^\alpha (e^{-p})^{\beta }. \end{aligned}$$

Therefore, for mutational counts, the model in (3) is equivalent to

4 $$\begin{aligned} \begin{aligned} V_{nm}|&p \sim \text{Po}( \tau p) \\&p \sim \text{Gamma}(\alpha , \beta ). \end{aligned} \end{aligned}$$

Since the Negative Binomial model is a Gamma–Poisson model we can also write the model as

$$\begin{aligned} V_{nm} \sim \text{NB} \left( \alpha , \frac{\tau }{\beta + \tau }\right) \backsimeq \text{NB} \left( \alpha , \frac{ \tau \mathbb{E}[ p]}{\alpha + \tau \mathbb{E}[p]}\right) \backsimeq \text{NB}\left( \alpha , \frac{(WH)_{nm}}{\alpha + (WH)_{nm}} \right) ,\end{aligned}$$

where the last parametrization is equivalent to the one in [18]. In the first distributional equivalence we use $\mathbb{E}[p] \approx \frac{\alpha }{\beta }$ and in the second we use $\tau \mathbb{E}[ p] = (WH)_{nm}$. Compared to the Beta-Binomial model, the Negative Binomial model has one fewer parameter and is analytically more tractable. The mean and variance of this model are given by

5 $$\begin{aligned} \mathbb{E}[V_{nm}] = (WH)_{nm} \quad \text{and} \quad \text{Var}(V_{nm}) = (WH)_{nm} \left( 1 + \frac{(WH)_{nm}}{\alpha }\right) . \end{aligned}$$

When $\alpha \rightarrow \infty$ above, the Negative Binomial model converges to the more commonly used Poisson model as $\text{Var}(V_{nm}) \downarrow (WH)_{nm}$. As shown in this section, the Negative Binomial model can be seen both as an extension of the Poisson model and as equivalent to the Beta-Binomial model. Thus, we opted to implement a Negative Binomial NMF model for mutational count data. More details on the approximation of the Negative Binomial to the Beta-Binomial distribution can also be found in [37].

### Patient specific NB_N_-NMF

In this section we describe our patient specific Negative Binomial non-negative matrix factorization NB_N_-NMF model and the corresponding estimation procedure.

Gouvert et al. [18], Lyu et al. [20] and Vöhringer et al. [21] present a Negative Binomial model where $\alpha$ is shared across all observations. However, the probability of a mutation in (3) is highly variable across patients (see e.g. mutational burden in [28] and our discussion in “Breast cancer data” section), thus we extend the Negative Binomial NMF model from [18] by allowing patient specific dispersion. We noticed that the variability among different patients is usually much higher than the one among different mutation types, thus we decided to focus on patient specific dispersion.

The entries in *V* are modeled as

$$\begin{aligned}V_{nm} \sim \text{NB}\left( \alpha _n, \frac{(WH^T)_{nm}}{\alpha _n + (WH^T)_{nm}}\right) , \end{aligned}$$

where $\alpha _n$ is the dispersion coefficient of each patient, and the corresponding Gamma–Poisson hierarchical model can be rewritten as:

6 $$\begin{aligned}&V_{nm}|a_{nm} \sim \text{Po}(a_{nm}(WH)_{nm}) \nonumber \\&a_{nm} \sim \text{Gamma}(\alpha _n, \alpha _n). \end{aligned}$$

Here $a_{nm}$ is the parameter responsible for the variability in the Negative Binomial model. Note that ${\mathbb{E}}[a_{nm}] = 1$ and $\text{Var}(a_{nm}) = 1/\alpha _n$.

Now we can write the Negative Binomial log-likelihood function with patent specific $\alpha _n$

7 $$\begin{aligned} \ell (W,H;V)&= \sum _{n=1}^N \sum _{m=1}^M \Bigg \{ \log { \left( {\begin{array}{c}\alpha _n + V_{nm} - 1\\ \alpha _n\end{array}}\right) } + V_{nm} \log \left( { \frac{(WH)_{nm}}{\alpha _n + (WH)_{nm}}}\right) \nonumber \\&\quad + \alpha _n \log \left( { 1 - \frac{(WH)_{nm}}{\alpha _n + (WH)_{nm}} } \right) \Bigg \}, \end{aligned}$$

and recognize the negative of the log-likelihood function as proportional to the following divergence:

8 $$\begin{aligned} d_N(V||WH)&= \sum _{n=1}^N \left\{ \sum _{m=1}^M V_{nm} \log \left( \frac{V_{nm}}{ (WH)_{nm}}\right) - (\alpha _n + V_{nm}) \log \left( \frac{\alpha _n + V_{nm}}{\alpha _n + (WH)_{nm}} \right) \right\} \end{aligned}$$

assuming fixed $\alpha _1, \ldots , \alpha _N$. This is a divergence measure as $d_N(V||WH) = 0$ when $V = WH$ and $d_N(V||WH)>0$ for $V \ne WH$. We can show this by defining $g(t) = (V_{nm}+t)\log \left( { (V_{nm}+t)}/{((WH)_{nm}+t) }\right)$ and realize $d_N(V||WH) = g(0) - g(\alpha ) \ge 0$ because $g'(t)\le 0$ with equality only when $V = WH$. The term $$\log { \left( {\begin{array}{c}\alpha _n + V_{nm} - 1\\ \alpha _n\end{array}}\right) }$$ in the likelihood is a constant we can remove and then we have added the constants $V_{nm} \log (V_{nm})$, $\alpha _{n} \log (\alpha _{n})$ and $(V_{nm} + \alpha _n) \log (V_{nm} + \alpha _n)$.

Following the steps in [18], we will update *W* and *H* one at a time, while the other is assumed fixed. We will show the procedure for updating *H* using a fixed *W* and its current value $H^t$. First we construct a majorizing function $G(H, H^t)$ for $d_N(V||WH)$ with the constraint that $G(H, H) = d_N(V||WH)$. The first term in Eq. (8) can be majorized using Jensen’s inequality leading to

9 $$\begin{aligned} d_N(V||WH)&= \sum _{n=1}^N \sum _{m=1}^M \Bigg \{ \{ V_{nm} \log \left( \frac{V_{nm}}{\sum _{k=1}^K W_{nk}H_{km}} \right) \nonumber \\&\quad - (\alpha _n + V_{nm}) \log \left( \frac{\alpha _n + V_{nm}}{\alpha _n + \sum _{k=1}^K W_{nk}H_{km}} \right) \Bigg \} \nonumber \\ {}&\le \sum _{n=1}^N \sum _{m=1}^M\Bigg \{ V_{nm} \log V_{nm} - V_{nm} \sum _{k=1}^K \beta _{k} \log \frac{W_{nk}H_{km}}{\beta _{k}} \nonumber \\ {}&\quad + (\alpha _n + V_{nm}) \log \left( \frac{\alpha _n + \sum _{k=1}^K W_{nk}H_{km}}{\alpha _n + V_{nm}} \right) \Bigg \} \end{aligned}$$

where $\beta _{k} = {W_{nk}H_{km}^t}/{\sum _{k=1}^K W_{nk}H^t_{km}}$. The second term can be majorized with the tangent line using the concavity property of the logarithm:

10 $$\begin{aligned} d_N(V||WH)&= \sum _{n=1}^N \sum _{m=1}^M \Bigg \{ V_{nm} \log V_{nm} - V_{nm} \sum _{k=1}^K \beta _{k} \log \frac{W_{nk}H_{km}}{\beta _{k}} \nonumber \\ {}&\quad + (\alpha _n + V_{nm}) \log \left( \frac{\alpha _n + \sum _{k=1}^K W_{nk}H_{km}}{\alpha _n + V_{nm}} \right) \Bigg \} \nonumber \\ {}&\le \sum _{n=1}^N \sum _{m=1}^M \Bigg \{ V_{nm} \log V_{nm} - V_{nm} \sum _{k=1}^K \beta _{k} \log \frac{W_{nk}H_{km}}{\beta _{k}} \nonumber \\ {}&\quad + (\alpha _n + V_{nm}) \log \left( \frac{\alpha _n + (WH^t)_{nm}}{\alpha _n + V_{nm}} \right) \nonumber \\ {}&\quad + \frac{W_{nm}}{\alpha _n + (WH^t)_{nm}}(H_{nm} - H^t_{nm}) \Bigg \} = G(H, H^t). \end{aligned}$$

Lastly, we need to show that $G(H, H) = d_N(V||WH)$. This follows from

11 $$\begin{aligned} G(H, H)&= \sum _{n=1}^N \sum _{m=1}^M \Bigg \{ V_{nm} \log V_{nm} - V_{nm} \sum _{k=1}^K \beta _{k} \log \frac{W_{nk}H_{km}}{\beta _{k}} \nonumber \\&\quad + (\alpha _n + V_{nm}) \log \left( \frac{\alpha _n + (WH)_{nm}}{\alpha _n + V_{nm}} \right) + \frac{W_{nm}}{\alpha _n + (WH)_{nm}}(H_{nm} - H_{nm}) \Bigg \} \nonumber \\&= \sum _{n=1}^N \sum _{m=1}^M \Bigg \{ V_{nm} \log V_{nm} - V_{nm}\sum _{k=1}^K \frac{W_{nk}H_{km}}{\sum _{k=1}^K W_{nk}H_{km}} \log \frac{W_{nk}H_{km}}{\frac{W_{nk}H_{km}}{\sum _{k=1}^K W_{nk}H_{km}}} \nonumber \\&\quad - (\alpha _n + V_{nm}) \log \left( \frac{\alpha _n + V_{nm}}{\alpha _n + \sum _{k=1}^K W_{nk}H_{km}} \right) \Bigg \} \nonumber \\&= \sum _{n=1}^N \sum _{m=1}^M \Bigg \{ V_{nm} \log V_{nm} - V_{nm} \cdot 1 \cdot \log \left( \sum _{k=1}^K W_{nk}H_{km} \right) \nonumber \\&\quad - (\alpha _n + V_{nm}) \log \left( \frac{\alpha _n + V_{nm}}{\alpha _n + \sum _{k=1}^K W_{nk}H_{km}} \right) \Bigg \} \nonumber \\&= \sum _{n=1}^N \sum _{m=1}^M \Bigg \{ V_{nm} \log \left( \frac{V_{nm}}{\sum _{k=1}^K W_{nk}H_{km}}\right) \nonumber \\&\quad - (\alpha _n + V_{nm}) \log \left( \frac{\alpha _n + V_{nm}}{\alpha _n + \sum _{k=1}^K W_{nk}H_{km}} \right) \Bigg \} \nonumber \\&= d_N(V||WH). \end{aligned}$$

Having defined the majorizing function $G(H, H^t)$ in Eq. (10), we can derive the following multiplicative update for *H*:

12 $$\begin{aligned} H_{km}^{t+1} = H_{km}^t \frac{\sum _{n=1}^N \frac{V_{nm}}{(WH^t)_{nm}} W_{nk}}{\sum _{n=1}^N \frac{V_{nm} + \alpha _n}{(WH^t)_{nm} + \alpha _n} W_{nk}}. \end{aligned}$$

Similar calculations can be carried out for *W* to obtain the following update:

13 $$\begin{aligned} W^{t+1}_{nk} = W^t_{nk} \frac{\sum _{m=1}^M \frac{V_{nm}}{(W^tH)_{nm}} H_{km}}{\sum _{m=1}^M \frac{V_{nm} + \alpha _n}{(W^tH)_{nm} + \alpha _n} H_{km}}. \end{aligned}$$

It is straightforward to see that when $\alpha _n = \alpha$ for all $n = 1, \ldots , N$ then the updates for *W* and *H* equal those in [18]. Additionally, as shown in [18] when $\alpha \rightarrow \infty$ the updates of the Po-NMF [14] are recovered.

In our application, we find maximum likelihood estimates (MLEs) of $\alpha _1, \ldots , \alpha _N$ based on the Negative Binomial likelihood using Newton–Raphson together with the estimate of *WH* from Po-NMF. We opted for this more precise estimation procedure for $\alpha _1, \ldots , \alpha _N$ instead of the grid search approach used in [18]. Final estimates of *W* and *H* are then found by minimizing the divergence in Eq. (8) by the iterative majorize-minimization procedure. The $\hbox{NB}_\text{N}$-NMF procedure is described in Algorithm 1 below. The model in [18, 20] is similar except $\alpha _1 = \cdots = \alpha _N = \alpha$.

It is well known that NMF can result in non-unique solutions [38]. Following these findings on the non-uniqueness and the effect of different initializations, all our results are based on five random initializations for each NMF solution.
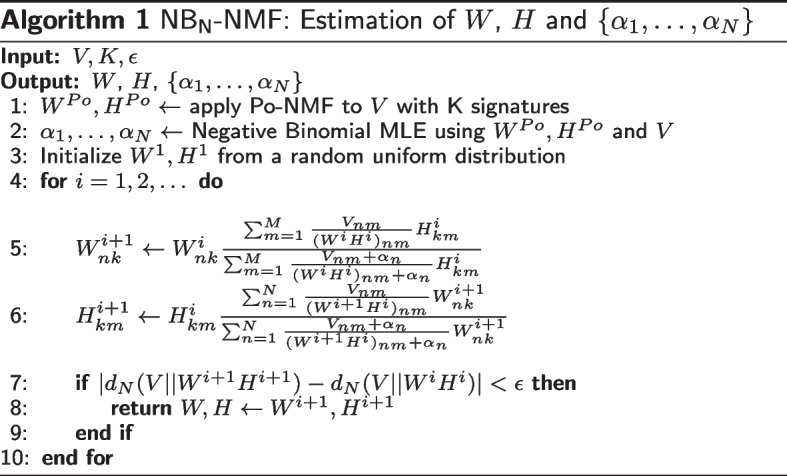


### Estimating the number of signatures

Estimating the number of signatures is a difficult problem when using NMF. More generally, estimating the number of components for mixture models or the number of clusters is a well known challenge in applied statistics.

Examples of the complexity of this problem can be found in the *K*-means clustering algorithm and in Gaussian mixture models where the number of clusters K has to be provided for the methods. A detailed description of these challenges can be found in [39]. Estimating the number of components is also a critical issue for mixed membership models. Some examples can be found in [40, 41].

Classical procedures to perform model selection are the AIC

14 $$\begin{aligned} \text{AIC} = -2\ln L + 2n_{prm} \end{aligned}$$

and the Bayesian Information Criterion (BIC)

15 $$\begin{aligned} \text{BIC} = -2\ln L + \ln (n_{obs}) n_{prm} \end{aligned}$$

where $\ln L$ is the estimated log-likelihood value, $n_{obs}$ is the number of observations and $n_{prm}$ the number of parameters to be estimated. The two criteria attempt to balance the fit to the data (measured by $-2\ln L$) and the complexity of the model (measured by the scaled number of free parameters). We have $n_{obs} = \text{N}$ where N is the number of patients, so $\ln (n_{obs}) > 2$ if $\text{N} \ge 8$, which means that in our context the number of parameters has a higher influence for BIC compared to AIC because real data sets always have at least tens of patients. Additionally, the structure of the mutational matrix *V* can lead to two different strategies for choosing $n_{obs}$ when BIC is used. Indeed, the number of observations in this context can be set as the total number of counts (i.e.  $N \cdot M$) or as the number of patients N, leading to an ambiguity in the definition of this criterion. Verity and Nichols [41] also presents results on the performance of AIC and BIC, where the power is especially low for BIC. AIC provides higher stability in the scenario from [41], however it does not seem suitable in our situation due to a small penalty term.

A very popular model selection procedure is cross-validation. In Gelman et al. [42] they compare various model selection methods including AIC and cross-validation. Here, the authors recommend to use cross-validation as they demonstrate that the other methods fail in some circumstances. In Luo et al. [43] they also show that cross-validation has better performance than the other considered methods, including AIC and BIC. Both papers evaluate the predictive fit to compare different methods.

#### Model selection for NMF

For NMF we propose an approach for estimating the rank which is highly inspired by cross-validation. As for classical cross-validation we split the patients in *V* in a training and a test set multiple times.

Since all the parameters in the model i.e. *W* and *H* are free parameters it means that the exposures for the patients in the test set are unknown from the estimation of the training set. The patients in the training set give an estimation of the signatures and the exposures of the patients in the training set. One could argue to fix the signatures from the training set and re-estimate exposures for the test set, but we observed that this lead to an overestimation of the test set.

Instead we have chosen to fix the exposures to the ones estimated from the full data. This means our evaluation on the test set is a combination of estimated signatures from the training set and exposures from the full data. The idea is to exploit the fact that the signature matrix should be robust to changes in the patients included in the training set. If the estimated signatures are truly explaining the main patterns in the data, then we expect the signatures obtained from the training set to be similar to the ones from the full data. Therefore the product of the exposures from the full data and the signatures from the training set should give a good approximation of the test set, if the number of signatures is appropriate. We tested this assumption on a real data set with hypermutated patients which may lead to patient specific signatures in Additional file 1: Section S3 and we find that our method is robust to the removal of the hypermutated patient.

Inputs for the procedure are the data *V*, an NMF method, the number of signatures K, the number of splits into training and test *J* and the *cost* function. We evaluate the model for a range of values of K and then select the model with the lowest cost. The NMF methods we are using here are either Po-NMF from [14] or $\hbox{NB}_\text{N}$-NMF in Algorithm 1, but any NMF method could be applied.

**Fig. 6:**
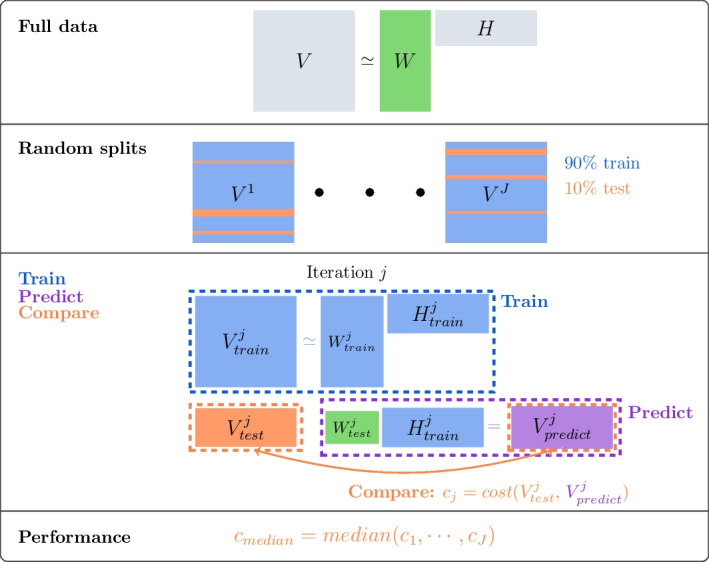
SigMoS procedure for a given number of signatures K and a count matrix *V*. Pseudocode can be found in Algorithm 2

A visualization of our model selection algorithm can be found in Fig. 6. First, we consider the full mutational matrix *V* and we apply the chosen NMF algorithm to obtain an estimate for both *W* and *H*. Afterwards, for each iteration, we sample 90% of the patients randomly to create the training set and determine the remaining 10% as our test set. We then apply the chosen NMF method to the mutational counts of the training set obtaining an estimate $W_{train}$ and $H_{train}$.

Now, as for classical cross-validation, we want to evaluate our model on the test set. To evaluate the model here, we use the full data: indeed, we multiply the exposures relative to the patients in the test set estimated on the full data $W_{test}^j$ times the corresponding signatures estimated from the training set $H_{train}^j$. As the order of the estimated signatures from the full data can be different to the one in the training set we reorder the exposures in $W_{test}^j$ with respect to the signatures in $H_{train}^j$. We determine the order by calculating the cosine similarity between the signatures in $H_{train}^j$ and those in *H*. We use the prediction of the test data to evaluate the model computing the distance between the true data $V_{test}^j$ and their prediction $V_{predict}^j$with a suitable *cost* function. This procedure is iterated *J* times leading to *J* cost values $c_j$, $j=1, \ldots , J$. The median of these values is calculated for each number of signatures K. We call this procedure SigMoS and summarize it in Algorithm 2. The optimal K is the one with the lowest cost. We use the generalized Kullback–Leibler divergence as a cost function and discuss the choice of cost function in “Discussion” section. We compare the influence of the model choice for our procedure to AIC and BIC. We also compare to $\texttt{SigProfilerExtractor}$, $\texttt{SignatureAnalyzer}$, $\texttt{SigneR}$ and $\texttt{SparseSignatures}$ as these are recently introduced methods in the literature and examine the results from this comparison in “Simulation study” section.
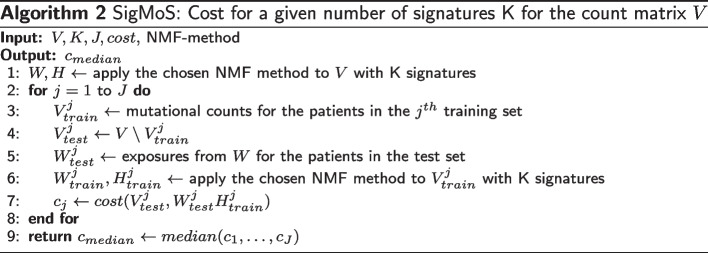


### Code for method comparison

For $\texttt{SigProfilerExtractor}$ we used the $\texttt{SigProfilerExtractor}$ Python package with $\mathtt {minimum\_signatures}$ equal to two, $\mathtt {maximum\_signatures}$ equal to eight and $\mathtt {opportunity\_genome}$ equal to “GRCh37”. For $\texttt{SparseSignatures}$ we use the function $\texttt{nmfLassoCV}$ with $\mathtt {normalize\_counts}$ being set to FALSE and $\mathtt {lambda\_values\_alpha}$ and $\mathtt {lambda\_values\_beta}$ to zero. All the other parameters are set to their default values. When applying $\texttt{SignatureAnalyzer}$ we used the following command $\texttt{python}$ $\mathtt {SignatureAnalyzer\text{- }GPU.py \quad \text{- }\text{- }data \quad f \quad \text{- }\text{- }prior\_on\_W \quad L1}$ $\mathtt {\text{- }\text{- }prior\_on\_H \quad L2 \text{- }\text{- }output\_dir \quad d \quad \text{- }\text{- }max\_iter \quad 1000000 \quad \text{- }\text{- }tolerance \quad 1e-7}$ $\mathtt { \text{- }\text{- }K0 \quad 8}$. For $\texttt{SigneR}$ we used the default options.

## Supplementary Information

**Additional file 1**. Supplementary material.

## Data Availability

The code for our model selection procedure and Negative Binomial NMF and for the simulations is available in the R package SigMoS and can be found at https://github.com/MartaPelizzola/SigMoS. The real data used in “Breast cancer data” section are available at ftp://ftp.sanger.ac.uk/pub/cancer/AlexandrovEtAl from [11]. The real data used in “Prostate cancer data” section can be found at https://www.synapse.org/#!Synapse:syn11726620.
