# Supplementary material for: Model selection and robust inference of mutational signatures using Negative Binomial non-negative matrix factorization
Source: BMC Bioinformatics. 2023 May 8;24:187. doi: 10.1186/s12859-023-05304-1 (PMC10165836; doi:10.1186/s12859-023-05304-1)
Supplement: Supplementary file 1 — Additional file 1. Supplementary material. [file 12859_2023_5304_MOESM1_ESM.pdf]

Supplementary material

S1 Results from simulated data in [30]

In this section we provide results from applying SigMoS to the data in [30]. Figures S1 and S2 show the ability of SigMoS to recover the true mutational signatures using Po-NMF and NB_N-NMF respectively.

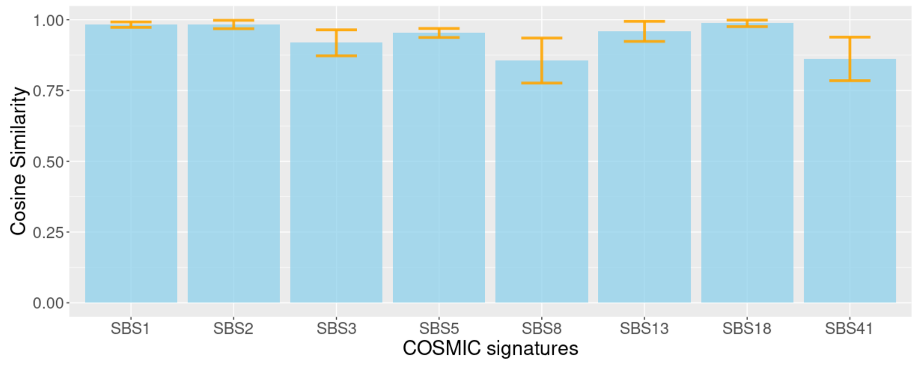

Figure S1 Method comparison using simulated data from [30]. Each method is applied on 20 data sets. For each data set the cosine similarity between the true signatures and the signatures estimated by SigMoS with Po-NMF is displayed. The height of the bars represent the mean of the 20 datasets and the yellow lines represent the standard deviation.

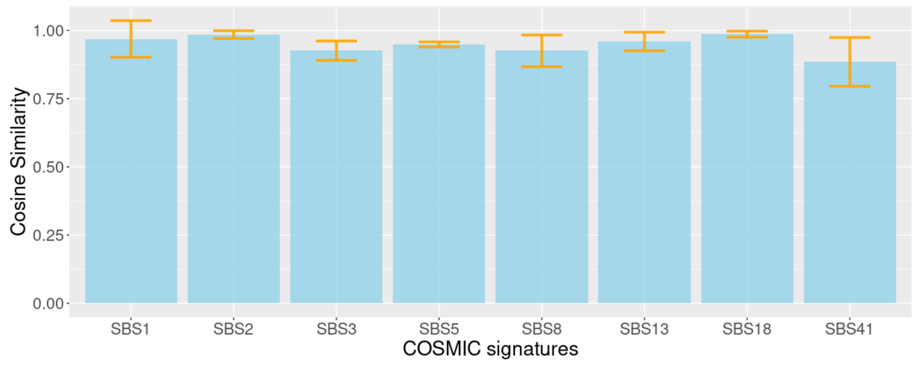

Figure S2 Method comparison using simulated data from [30]. Each method is applied on 20 data sets. For each data set the cosine similarity between the true signatures and the signatures estimated by SigMoS with NB_N-NMF is displayed. The height of the bars represent the mean of the 20 datasets and the yellow lines represent the standard deviation.

S2 Computational cost for SigMoS with Po-NMF and NB_N-NMF

In this section we present results on the memory consumption and running time for SigMoS using Po-NMF and NB_N-NMF as a function of the number of patients. We simulated data following the model in Section 2.1 and varying the number of patients in $\{20, 50, 100, 150, 200, 500, 1000\}$. For each of these scenarios we run 20 simulations with 5 signatures. SigMoS is running in parallel for $J = 100$ iterations of the model selection algorithm (see Figure 6). The memory consumption can thus be decreased by a fraction a in exchange for an increase in time by the corresponding multiplicative factor a. All analysis have been conducted on a standard laptop with Intel Core i7 processor.

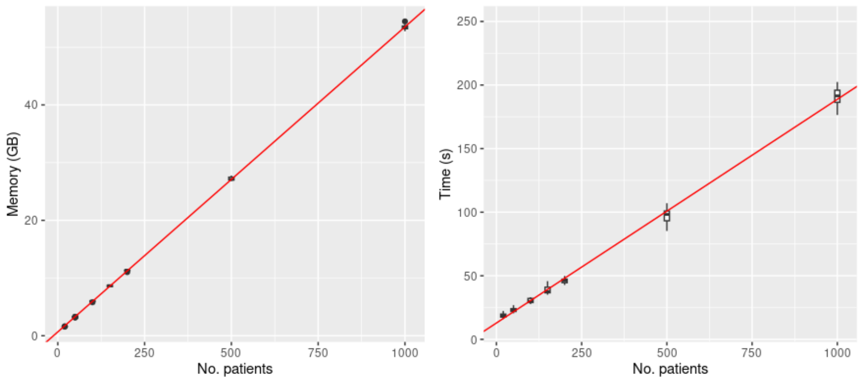

Figure S3 Memory consumption in gigabytes (GB) and running time in seconds (s) for SigMoS with Po-NMF with 5 signatures as a function of the number of patients. The tolerance for convergence is set to 10^{-5} and 100 iterations of cross-validation are performed. The boxplots show results for 20 runs of SigMoS for each number of patients.

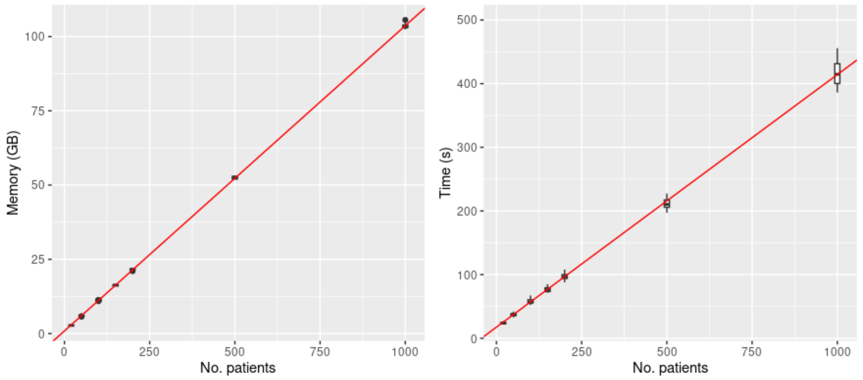

Figure S4 Memory consumption in gigabytes (GB) and running time in seconds (s) for SigMoS with NB_N-NMF with 5 signatures as a function of the number of patients. The tolerance for convergence is set to 10^{-5} and 100 iterations of cross-validation are performed. The boxplots show results for 20 runs of SigMoS for each number of patients.

S3 Influence of hypermutated patients

We considered the real data set from [6] and applied both SigMoS with Po-NMF and with NB_N-NMF. First, we applied both methods to the 21 patients included in the data, and then we applied the same methods excluding the hypermutated patient (the last patient in the data). Figures S5 and S6 show the estimated mutational signatures from the Poisson and Negative Binomial models, respectively, and the normalized mutational profile of the hypermutated patient. The correlation between the profile of the hypermutated patients and the extracted signatures is (0.3002, 0.9999, 0.3203) with Po-NMF and (0.2135, 0.9999, 0.2013) under the Negative Binomial model.

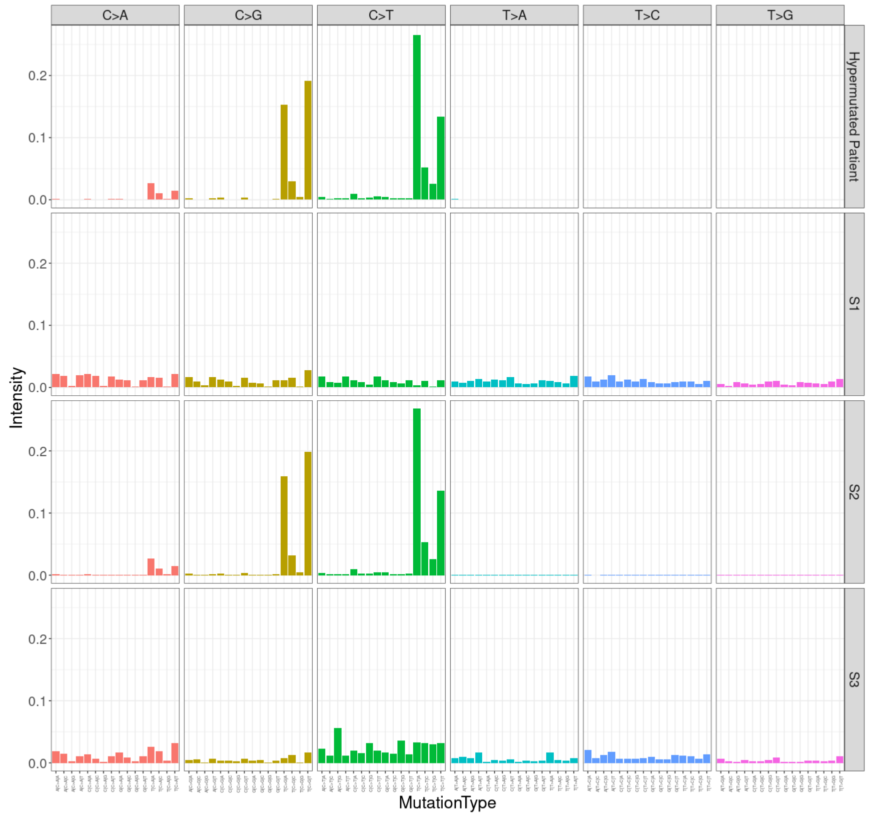

Figure S5 Signatures extracted from SigMoS with Po-NMF using the 21 breast cancer patients from [6] and mutational profile of the hypermutated patient in this data set.

When removing the hypermutated patient and running SigMoS again we obtain very similar signatures. The correlation between the hypermutated patient and the signatures indeed is (0.2453, 0.9894, 0.3095) under the Poisson model and (0.2755, 0.9903, 0.2906) with NB_N-NMF. This shows that the estimation of the signatures with SigMoS is not affected by the hypermutated patient and that our method is robust in this setting. It appears that the mutations of the hypermutated patient indeed originate from signature S2 that is shared among other patients as well. Thus our assumptions in the SigMoS method are not violated and the signatures can be correctly estimated also in this scenario.

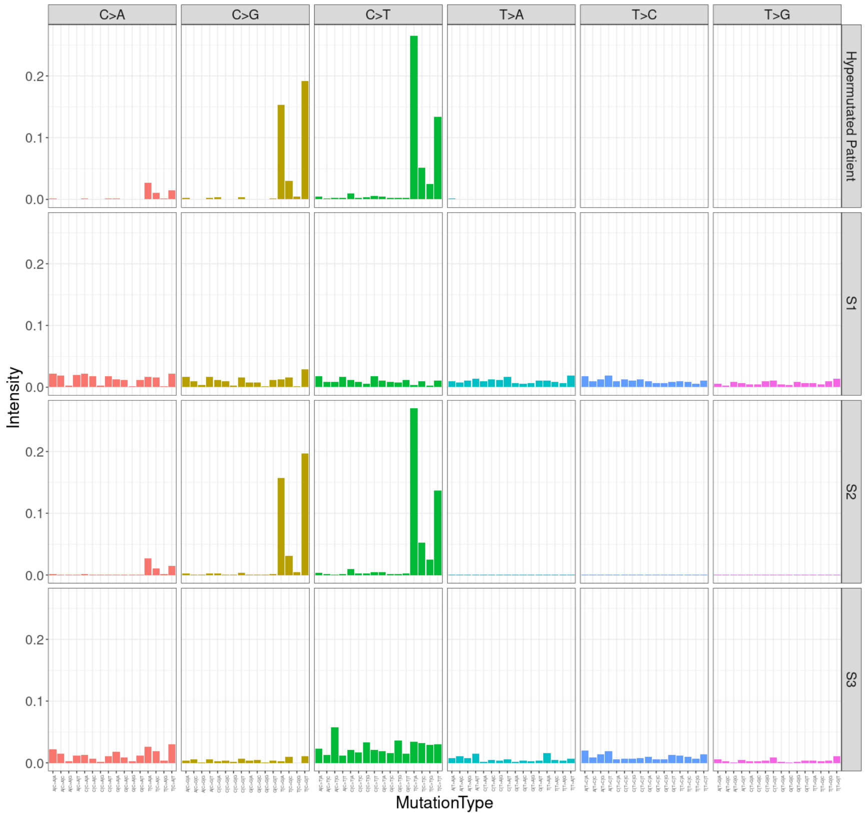

Figure S6 Signatures extracted from SigMoS with NB_N-NMF using the 21 breast cancer patients from [6] and mutational profile of the hypermutated patient in this data set.

Figures S7 and S8 show the extracted exposures with Po-NMF and NB_N-NMF respectively. The top panels show results with all patients and the lower panels show results when the hypermutated patient is removed. This also emphasizes that the exposures to the reconstructed signatures are very similar (note the difference in the y-axis scale) in the two settings, suggesting a robust signature extraction.

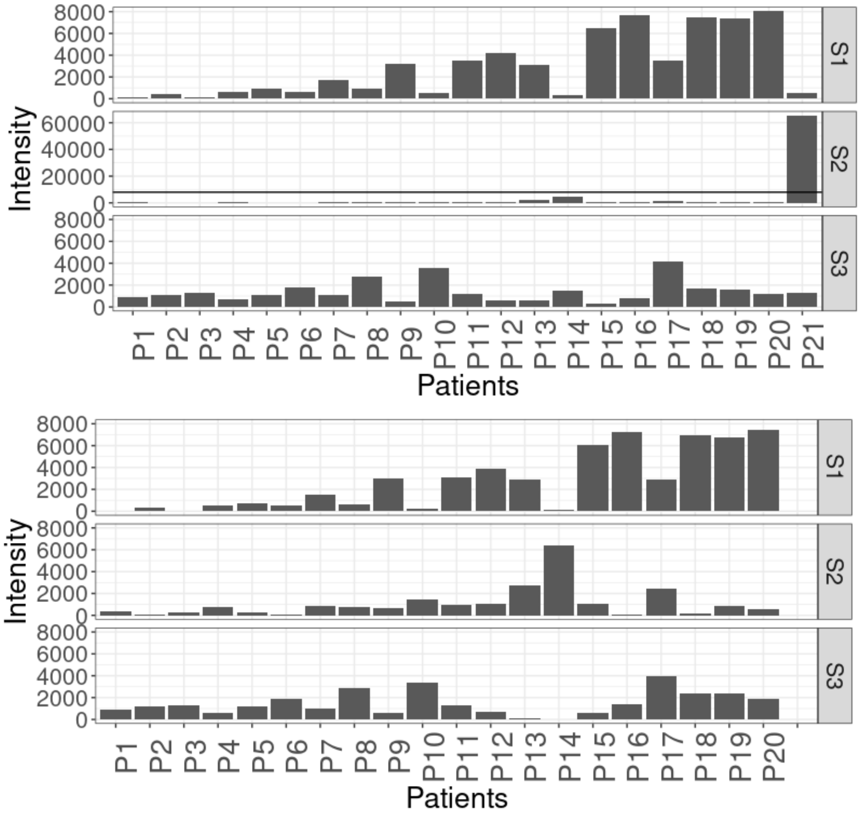

Figure S7 Exposures extracted from SigMoS with Po-NMF using the 21 breast cancer patients from [6] (top panel) and excluding the hypermutated patient (bottom panel). The horizontal black line in the top panel corresponds to an intensity equal to 8000 for comparison with the lower panel.

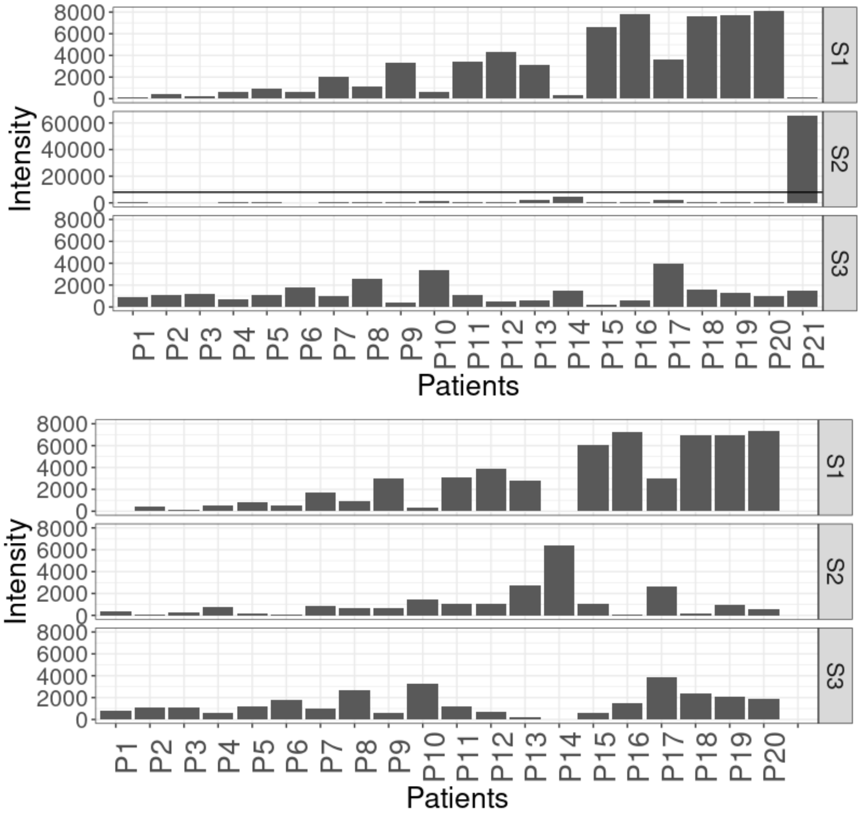

Figure S8 Exposures extracted from SigMoS with NB_N-NMF using the 21 breast cancer patients from [6] (top panel) and excluding the hypermutated patient (bottom panel). The horizontal black line in the top panel corresponds to an intensity equal to 8000 for comparison with the lower panel.

S4 Influence of the choice of the cost function on SigMoS

We note that the choice of the divergence measure for the cost function in Algorithm 2 is not trivial and may favor one or the other model. For example, in our framework, we may be biased towards the Poisson model when using the Kullback-Leibler (KL) divergence. Here, we test the influence of the choice of the cost functions in the SigMoS procedure. We consider the Itakura-Saito (IS) [32] divergence and the Frobenius norm as alternative cost functions for our proposed cross-validation algorithm. Both cost functions are also implemented in the SigMoS R package.

The KL divergence is given by equation (8) with $\alpha_1 = \cdots = \alpha_N = 0$. Using the same notation as in Algorithm 2 with $B = V^j_{test}$ and $B^0 = H^j_{train} W^j_{test}$ the IS divergence can be expressed as:

$$d_{IS}(B, B^0) = \sum_{n=1}^{N} \sum_{m=1}^{M} \left\{ \frac{B_{nm}}{B^0_{nm}} - \log \frac{B_{nm}}{B^0_{nm}} \right\}.$$

And the Frobenius norm is given by:

$$d_{FB}(B, B^0) = \sum_{n=1}^{N} \sum_{m=1}^{M} (B_{nm} - B^0_{nm})^2.$$

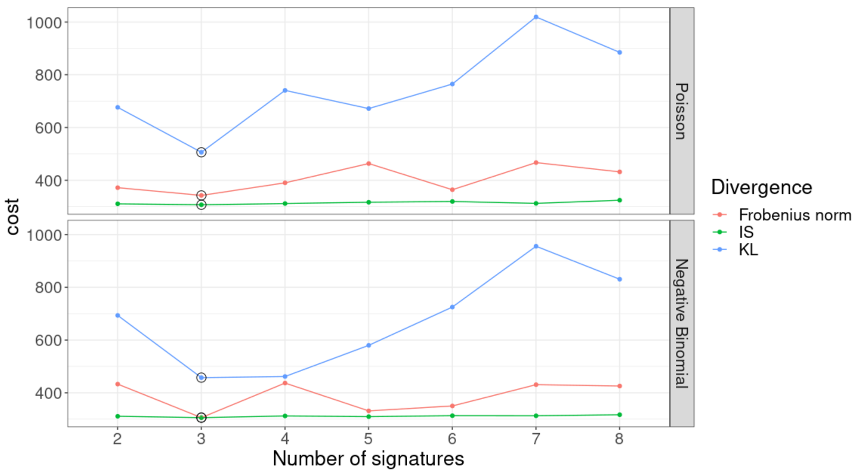

Figure S9 Cost function analysis. The cost from SigMoS as a function of the number of signatures. SigMoS is applied to 21 breast cancer patients from Section 2.2 using the Frobenius norm, the Itakura-Saito and the Kullback-Leibler divergence as cost functions for both Po-NMF and NB_N-NMF.

We test our model selection procedure with the KL, IS and the Frobenius norm. We test the influence of the choice of the cost function on the same data described in Section 2.2 corresponding to the 21 breast cancer patients in [6]. The results of this analysis are shown in Figure S9 and demonstrate that our model selection framework is robust to different choices of the cost function. However, we observe that the minimum is more pronounced when using the KL divergence.
